# Supplementary material for: A random optical parametric oscillator
Source: Nat Commun. 2023 Oct 20;14:6664. doi: 10.1038/s41467-023-42452-7 (PMC10589305; doi:10.1038/s41467-023-42452-7)
Supplement: Supplementary file 1 — Supplementary Information [file 41467_2023_42452_MOESM1_ESM.pdf]

Supplementary Material

A Random Optical Parametric Oscillator

Supplementary Notes

As mentioned in the main text, the coherence factor ξ must be taken into account for the calculation of the ROPO's threshold. This factor represents how much above the mean reflectivity (or incoherent case) can we expect the highest reflectivity peak to be found. Based on the theoretical model developed in [28], we numerically calculate the intensity fluctuations of Rayleigh backscattered coherent light in SMFs. In the model, the fibre is pictured as a sequence of small sections d of constant average refractive index, which changes randomly from section to section due to a fabrication artefact. The backscattered optical field E_b at the launching end ($z = 0$) of an SMF was described as a function of the wavenumber k by

$$E_b(k) = E_0 S \sum_{i=1}^{N} E_i, \tag{1}$$

$$\text{where, } E_i = \exp(j2\phi_{i-1}) \int_{-d/2}^{d/2} \exp(-\alpha(l + z_i) - j2\bar{n}_i k l)\, dl$$

$$\text{and } \phi_i = \phi_{i-1} + \bar{n}_i k d.$$

In Supplementary Equation (1) above, S is the Rayleigh capturing coefficient, E_0 is the input optical field, α is the attenuation coefficient, $\bar{n}_i$ is the mean refractive index for the ith fibre section, ϕ_i is the phase accumulated up to the ith section, and N is the total number of sections, such that the fibre length L is simply $N \cdot d$. The power reflectivity for each optical frequency $\nu = ck/2\pi$ is calculated through $|E_b(\nu)/E_0|^2$, and strong reflectivity fluctuations are predicted as a function of optical frequency [28], similar to the jagged reflectivity profile of random gratings [29]. Here, rather than considering the whole fibre in the calculation, we modify Supplementary Equation (1) to include only an array of short fibre sections with length defined by the pulse width W and positions determined from Equation (1) of the main text. The modified backscattering field is given by

$$E_b(z = 0, k) = E_0 S \sum_{i=1}^{N} E_i p_i \tag{2}$$

$$p_i = \begin{cases} 1 & ; \; z_m \leq z_i \leq z_m + W \\ 0 & ; \; \text{otherwise} \end{cases}$$

We performed the numeric calculation of Supplementary Equation (2) using the parameters of the fibre used in experiments, a pulse repetition rate of 1 MHz, and a pulse duration of 5 ns. The power reflectivity was then calculated for an optical frequency range of 10 GHz, corresponding to the ten percent flat top bandwidth of the FBG. Indeed, the result is similar to that reported in [28], and an example is shown in Supplementary Fig. 1. The highest reflectivity peak is 10.4 times higher than the mean value. Repeating the numerical calculation twenty times, the highest peak obtained is on average about 10 times higher than the mean value with a maximum deviation of $\sim$10 %, justifying the usage of $\xi = 10 \pm 1$ in the calculation of ROPO threshold (Equation (3) and Fig. 3d in the main text).

Supplementary Methods

We characterised the reflectivity from Rayleigh scattering in piecewise distributed sections of the 5.25 km-long fibre by launching 5 ns pulses and varying the pulse repetition rate from 100 kHz to 1 MHz. The measurement setup, displayed in Supplementary Fig. 2, was developed with a gated synchronous transmission/detection scheme, reproducing the feedback of a discrete number of fibre sections synchronised

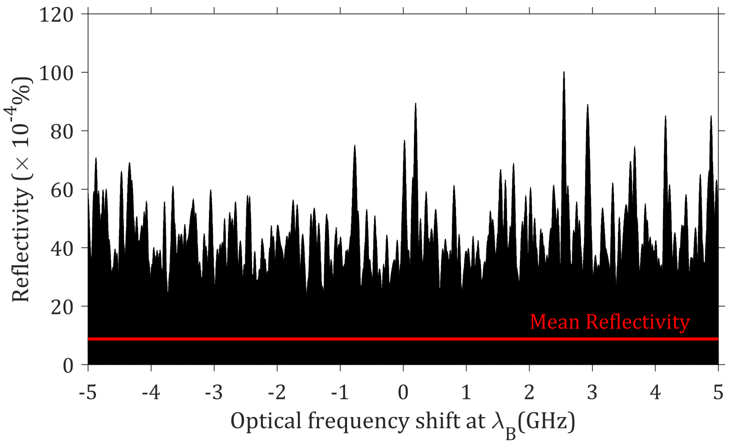

Supplementary Figure 1: **Spectral reflectivity profile**. Numerical calculation result for the reflectivity of piecewise distributed fibre sections. Total fibre length was 5.25 km, and each effective Rayleigh section was designed with 0.5 meter (from 5 ns pulses) spaced from other sections by about 100 meters (repetition rate of 1 MHz).

with incoming pulses. Light from a broadband source (∼20 nm) was filtered (1 nm) and amplified (EDFA) before modulated with 5 ns square pulses by an SOA. The filter was required to allow stronger amplification before launching, so that the optical pulses carried high power while still sufficiently incoherent. Pulses were launched through a circulator into the SMF with 5.25 km. We bent the fibre end with a short radius curve to prevent a strong reflection at the connector. Backscattered light collected at the circulator was amplified and modulated at an electro-optic modulator with 5 ns pulses synchronised with those driving the SOA, all controlled from an arbitrary waveform generator (AWG). A time-delay was carefully set to the pulses driving the EOM to equalise the fibre pigtails from the SOA and EOM. By modulating the detected signal, we investigate the backscattered light from multiple fibre sections that meet an incoming pulse at the launching end. A high-gain photodetector (PD) was used for detection of the gated backscattered signal. The result is shown in Fig. 3a-b (main text).

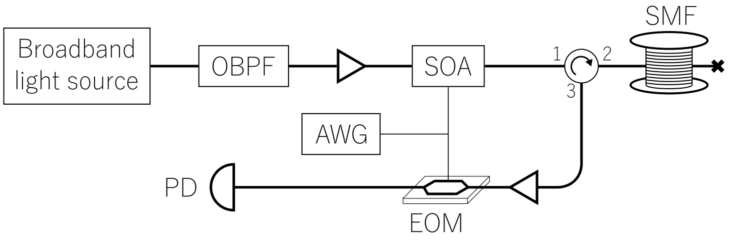

Supplementary Figure 2: **Reflectivity characterisation setup**. Experimental setup for the characterisation of the reflectivity of piecewise distributed fibre sections. AWG: Arbitrary waveform generator; EOM: Electro-optic modulator; OBPF: Optical band-pass filter; PD: photodetector; SMF: Single-mode fibre; SOA: Semiconductor optical amplifier.
